# Dynamics of the Novel Cardiac Biomarkers sST2, H-FABP, GDF-15 and suPAR in HFrEF Patients Undergoing Heart Failure Therapy, a Pilot Study

**DOI:** 10.3390/jcm14165668

**Published:** 2025-08-11

**Authors:** Bernhard Ohnewein, Zornitsa Shomanova, Peter Jirak, Vera Paar, Albert Topf, Lidia Pylypenko, Max Schäbinger, Fabian Volg, Uta C. Hoppe, Rudin Pistulli, Naufal Zagidullin, Michael Lichtenauer, Lukas J. Motloch

**Affiliations:** 1 Department of Internal Medicine II, Paracelsus Medical University, 5020 Salzburg, Austrialukas.motloch@ooeg.at (L.J.M.); 2 Department of Cardiology, University Hospital Münster, 48149 Münster, Germany; 3 Rehabilitation Center Moorheilbad Harbach, 3970 Moorbad Harbach, Austria; 4 Rehabilitation Center Lebens.Resort Ottenschlag, 3631 Ottenschlag, Austria; 5 Department of Internal Diseases, Bashkir State Medical University, 450008 Ufa, Russia; znaufal@mail.ru; 6 Department of Cardiology, Kepler University Hospital, Johannes Kepler University, 4020 Linz, Austria; 7 Department of Internal Medicine II, Salzkammergut Klinikum, OÖG, 4840 Vöcklabruck, Austria

**Keywords:** neprilysin, angiotensin, inhibition, heart failure, biomarkers, sST2, GDF-15, H-FABP, suPAR, HFrEF, ARNI

## Abstract

**Background:** Despite improvements in medical therapy, heart failure with reduced ejection fraction (HFrEF) is a major burden on the healthcare system and remains a leading cause of death with a 5-year mortality rate of more than 60%. Novel therapeutic agents such as angiotensin-receptor-neprilysin-inhibitors (ARNIs) lead to significant improvement in clinical outcomes. Optimal therapy monitoring under these novel drugs is crucial for improving the outcome. In this trial, the diagnostic potential of four novel cardiovascular biomarkers—GDF-15, sST2, H-FABP, and suPAR—was evaluated during follow-up in patients with HFrEF. **Methods:** In this prospective cohort pilot study, 70 patients with HFrEF with ischemic (n = 34) and non-ischemic (n = 36) origin were included. All included patients were on a stable treatment regimen and in a non-decompensated state. The clinical parameters NYHA class, LVEF, MPI/Tei index and ESC Score 2 and the laboratory parameters sST2 (remodeling, inflammation), GDF-15 (remodeling, inflammation), H-FABP (subclinical ischemia and ischemia), suPAR (remodeling, inflammation) and NT-proBNP were assessed before ARNI therapy initiation and at 3 to 6 months at follow-up. Before starting ARNI therapy with sacubitril/valsartan patients had stable and well-established heart failure therapy. **Results:** There was a sufficient response to therapy with significant improvement in ejection fraction from 29.9% to 38.5% (*p* < 0.001) and a significant decrease in NT-proBNP from 1402 pg/mL to 572.0 pg/mL (*p* = 0.003). Interestingly, along with that, a significant increase in sST2 levels from 9602 pg/mL to 12,001 pg/mL (*p* = 0.039) but no significant change in H-FABP (*p* = 0.397), GDF-15 (*p* = 0.382) or suPAR (*p* = 0.328) were observed. Furthermore, the baseline sST2 level correlated with the risk of cardiovascular events calculated with the ESC Score 2 and the GDF15 level at follow-up correlated with the right ventricular global function, assessed with the MPI/Tei index and this correlation persisted after correction for confounders (*r* = 0.323, *p* = 0.039; *r* = 0.504, *p* = 0.011). **Conclusions:** The novel biomarker sST2 but not H-FABP, GDF-15 and suPAR was significantly affected by medical therapy with ARNIs. Monitoring sST2 might offer new opportunities for therapy guidance and disease management. However, these results are hypothesis generating and should be interpreted with caution, given the pilot nature of this study.

## 1. Introduction

The global prevalence of heart failure (HF) is approximately 64.3 million cases and is estimated to further increase by approximately 3% until 2030 [1,2]. Considering a 5-year mortality rate of more than 60% in heart failure with reduced ejection fraction (HFrEF), it is even more reasonable to define HF as a global pandemic [3].

Once diagnosed, patients are hospitalized due to HF approximately once a year. Consequently, it is the major cause of hospitalization in the population aged 65 years or older [4,5]. It is curious that despite improving heart failure therapy, the rate of first hospitalizations has been rising again since 1998 [6,7,8]. Along with the increasing morbidity and hospital admissions, the economic burden of HF, worth hundreds of billions of dollars a year, is rising [9]. Given this struggle of HF, sufficient patient monitoring is crucial to reduce hospital admissions and stress upon the healthcare system.

While current guidelines give detailed information about the diagnosis and treatment of HF, sufficient patient monitoring is still an important field of investigation. Promising trials in device monitoring showed the potential of improved follow-up in chronic HF. Device-based pulmonary artery pressure monitoring showed a significant reduction in HF hospitalizations in the US and Germany [10,11]. Results of a direct comparison to an intensive standard care in Germany are still awaited [12].

Numerous studies have highlighted the importance of biomarkers for the diagnosis and prognosis of HF [8]. With respect to the multiple entities of heart failure and numerous mechanisms aggravating heart failure the fact there is only one established biomarker in clinical routine is surprising. NT-proBNP elevation is correlated with a worse prognosis although a medical therapy guided by NT-proBNP levels did not show any benefit [13]. Coming to the conclusion that despite the significant dynamics in HF therapy, optimal monitoring parameters have not emerged yet [14,15]. This is aggravated by novel HF therapy agents, which in turn have an influence on the established biomarker brain natriuretic peptide (BNP) levels. As neprilysin inhibition by ARNIs decreases BNP metabolization the blood levels of BNP do no longer resemble the severity of HF.

Many novel biomarkers have shown significant correlation with HF; however, the impact of ARNIs on the concentrations of circulating biomarkers is not fully understood yet. In this study, we intend to gain better insight into the effect of (angiotensin-receptor-neprilysin-inhibitors) ARNIs on the novel HF biomarker.

Previous studies have shown that the biomarkers soluble suppression of tumorigenicity 2 (sST2), soluble urokinase plasminogen activator receptor (suPAR), heart-type fatty acid binding protein (H-FABP) and growth differentiation factor 15 (GDF-15) are significantly elevated in ischemic and dilatative cardiomyopathy compared to healthy controls [16]. Furthermore, GDF-15 and H-FABP have also been shown to significantly increase in the circulation of HFpEF patients [17].

The ST2 receptor was first identified in 1989 as a protein that interacts with fibroblasts and induces their proliferation [18]. Fifteen years later, its ligand interleukin-33 (IL-33) was identified [19]. ST2 is also a membrane protein in cardiac myocytes and its expression is predominantly upregulated during myocyte stretch [20]. The binding of IL-33 to the membrane-bound ST2 exerts anti-hypertrophic and antifibrotic effects on the myocardium. Interestingly, the role of IL-33 is not yet fully understood. Other trials have shown negative effects of IL-33, including the activation of inflammatory pathways. IL-33 induces the secretion of proinflammatory cytokines, an aggravation of atherosclerosis, and a promotion of endothelial dysfunction. However, these effects might also reflect reduced bioactivity due to its binding to the soluble form of ST2 (sST2). This protein might act as a decoy receptor and attenuates the IL-33 signaling pathway [21,22]. Increased levels of sST2 were previously associated with increased mortality in severe HF [23].

The protein GDF-15 was first described in 1997 as MIC-1 and is considered a member of the TGF-β superfamily due to its highly conserved seven-cysteine domain, although it exhibits divergent effects [24]. Since it was found in multiple tissues but is predominantly expressed in reproductive organs, the name GDF-15 was established, as proposed by Böttner et al. [25]. GDF-15 is upregulated during inflammation, pressure overload and ischemia and acts anti-apoptotic, anti-inflammatory and anti-hypertrophic [26,27]. Increased levels of GDF-15 might serve as a marker of pressure overload and subclinical ischemia in healthy individuals since they were associated with 2.7 fold increased risk of cardiovascular events [28,29,30].

The inflammatory marker suPAR came into focus since 1991 when it was found to have a strong affinity f the membrane bound form uPAR [31]. uPAR plays an important role in cell migration and inflammation during the adhesion process of inflammatory cells, such as monocytes and T-cells, which is attenuated by the soluble form suPAR [32]. To date, associations with cancer, inflammatory disease, and cardiovascular disease have been described [33,34,35,36]. Increased levels of suPAR are an independent predictor of mortality in HF patients [37].

Fatty acid-binding proteins are known since the 1970s to bind to long chain fatty acids and serve as transport proteins [38]. H-FABP is expressed in higher amounts in the cardiac muscle and diffuses into the blood in case of cardiac injury [39]. As it is a very early marker of cardiac damage, which is detectable in the blood even before troponin, it became of special interest as a cardiac biomarker [40]. Increased plasma levels of H-FABP are associated with higher rates of cardiovascular events, cardiovascular death, and all-cause mortality [41]. Interestingly, H-FABP was shown to detect patients at increased risk of death and non-fatal cardiac events, even when troponin was negative [42].

Heart failure is frequently associated with chronic inflammation. More than half of patients with HFrEF had elevated interleukin levels in the BIOSTAT-CHF study, and an even higher percentage of patients with HFpEF had elevated CRP levels [43,44]. Multiple mechanisms, such as ischemia and pressure overload, cause myocardial injury and initiate the release of cytokines and chemokines. This cascade triggers the infiltration of monocytes and neutrophils, which aggravates the inflammation through the further release of interleukins. As inflammation progresses, T cells and B cells migrate and enhance fibrotic processes. Specific pathophysiological mechanisms in heart failure, such as activation of the renin–angiotensin–aldosterone system and the sympathetic nervous system, further aggravate the inflammatory processes [22]. The impact of inflammation is reflected by the increased risk of mortality in heart failure. Patients with HFrEF and elevated CRP levels had a significantly increased risk of cardiovascular death and all-cause mortality. Similar results have been observed for markers of systemic inflammation in HFpEF, where the risk of all-cause mortality was increased by 1.43-fold, and the risk of cardiovascular mortality was increased by 2.02-fold [45].

Novel therapy agents like ARNIs lead to a significant decrease in mortality. ARNIs inhibit neprilysin and thereby increases circulating BNP levels, resulting in beneficial effects on HF by reducing preload, inflammation, and fibrosis [46,47]. Considering the multiple interaction pathways of ARNIs, including effects on natriuretic peptides, bradykinin, adrenomedullin, substance P, angiotensin I and II, endothelin, and even lipid and glucose metabolism parameters [48,49], it is of special interest whether the current knowledge of novel biomarkers also apply under ARNI therapy This might offer new insights into the effects of ARNIs on inflammatory pathways.

In this study, we aimed to investigate the impact of ARNIs on the dynamics of circulating biomarkers during HF therapy and thereby contribute to the development of a multi-biomarker strategy leading to a personalized medicine approach in future studies. We attempt to generate a better understanding of potential alterations in the biomarker concentrations by ARNI therapy in HF.

## 2. Materials and Methods

### 2.1. Study Population

In this prospective observational cohort pilot trial, we included patients from two university hospitals. This study adheres to the STROBE (Strengthening the Reporting of Observational Studies in Epidemiology) guidelines. A completed STROBE checklist is provided in the Supplementary Materials. In total, 70 patients with HFrEF of ischemic (n = 36) and non-ischemic (n = 34) origin were included. All patients presented at the University Hospital Muenster, Germany or University Hospital of Salzburg, Austria with newly diagnosed chronic HF or progression of chronic HF. The primary exposure was the initiation of angiotensin receptor–neprilysin inhibitor (ARNI) therapy sacubitril/valsartan titrated to the target dose of 97/103 mg twice daily or as high as tolerated until the follow-up date. The level of ARNI dose was evaluated at the follow up. All patients had a minimum dose of 24/26 mg twice daily. The target ARNI dose of 97/103 mg twice daily was reached on average by 69% of patients at follow-up. Inclusion criteria were typical symptoms, reduced left ventricular ejection fraction (LVEF) ≤ 40%, elevated N-terminal pro-brain natriuretic peptide (NT-proBNP) > 300 ng/L and planned start of treatment with ARNIs. All patients were on stable treatment regimens before initiation of ARNI therapy. In case of intolerance of ARNI therapy, patients were excluded. Subjects who provided informed consent were enrolled between March 2019 and December 2020.

### 2.2. Data Collection

Baseline clinical data—including demographics, medical history, heart failure etiology, and medication—were obtained through patient interviews and medical records at enrollment. Blood samples were taken during acute hospitalization and after three to six months of follow-up. Therefore, all patients had a scheduled follow-up appointment at the cardiology outpatient clinic. Samples were processed and stored under standardized conditions. Laboratory staff were blinded to clinical and treatment data to reduce measurement bias. Additionally, clinical investigations and echocardiography were performed. The primary outcome were the dynamics of NYHA class, LVEF, in addition to levels of sST2, suPAR, GDF-15 and H-FABP under therapy. Furthermore, we evaluated the correlation of those markers with the risk of cardiovascular events, calculated by the ESC Score 2 and the correlation with the right ventricular function, assessed with the myocardial performance index (MPI)/Tei index. No patient was lost during follow-up. Exclusion criteria were severe valvular heart disease, incompliance, or discontinuation because of severe side effects. The outcome variables were defined as changes in sST2, GDF-15, suPAR and H-FABP levels and were considered significantly affected if the *p*-values were below 0.05. To assess potential clinical relevance, a correlation with the risk predictor model “ESC Score 2” for prediction of fatal and nonfatal cardiovascular disease events and known relevant clinical parameters like left ventricular function (LVEF) and right heart function (MPI/Tei index) were calculated.

To minimize selection bias, patients were consecutively enrolled from two university hospitals based on predefined inclusion criteria, ensuring a representative sample of individuals with HFrEF eligible for ARNI therapy. To reduce information bias, standardized processes were used for clinical assessments and blood sample collection at baseline and follow-up. Laboratory personnel were blinded to clinical data and treatment status during biomarker analyses. Confounding was addressed by identifying potential confounders based on previous literature and clinical expertise, including age and comorbidities. These variables were adjusted for in multivariable regression models to isolate the effect of ARNI therapy on biomarker changes. Nevertheless, residual confounding from unmeasured variables cannot be fully excluded. As this study is as a pilot trial, no formal sample size calculation was performed prior to enrollment. The sample size of 70 patients was based on feasibility considerations, oriented on previous trials and aimed to provide preliminary data on biomarker changes following ARNI therapy in patients with HFrEF.

### 2.3. Blood Sampling

Blood was obtained from a cubical vein and collected in a serum tube to initiate blood coagulation. After 30 min, the blood was centrifuged at 2000× *g* at 4 °C for 20 min. The resulting supernatant was withdrawn and was immediately stored at −80 °C until further analysis.

### 2.4. Biomarker Analysis

Serum concentrations of sST2 (DY523B-05), suPAR (DY807), GDF-15 (DY957) and H-FABP (DY1678) were measured by commercially available enzyme-linked immunosorbent assays (ELISAs), purchased from R & D Systems (Abingon, UK). The assays were performed according to the manufacturer’s instructions.

In brief, 96-well plates were coated with a provided capture antibody prior to the incubation on an orbital shaker overnight. The next day, plates were washed three times with 1× phosphate buffered saline (PBS) with 0.05% Tween 20 solution (PBS-T, Carl Roth GmbH + Co. KG, Karlsruhe, Germany). Thereafter, plates were blocked for a minimum of 1 h by adding 300 µL of 1% bovine serum albumin (BSA) in 1× PBS. After a further washing step, an appropriate serial dilution of the provided standard and the serum samples were added to the precoated and blocked wells. After an incubation of 2 h, palates were washed again and 100 µL of a provided biotinylated detection antibody was applied, followed by a 2 h incubation under horizontal agitation. After a further washing step, streptavidin horseradish peroxidase (HRP) was added and incubated for 20 min in the dark. Following the final washing step, the colorimetric reaction was achieved by adding the tetramethylbenzidine (TMB) substrate (Merck KGaA, Darmstadt, Germany). Finally, the reaction was shopped by 50 µL of 2 N sulfuric acid solution (H_2_SO_4_, Merck, Germany) per well. The resultant yellow color was then measured by a microplate absorbance reader (iMark Microplate Reader, Bio-Rad Laboratories, Inc., Hercules, CA, USA) at 450 nm. The proteins’ concentrations were quantified automatically via Microplate Manager Software (MPM 6, product version: 6.3, Bio-Rad, Germany) by employing optical density (OD) and a defined standard curve.

### 2.5. Ethics

This study conformed to the Declaration of Helsinki and had ethics approval by the local ethics committee (protocol codes in Münster/Salzburg: 2019-011-f-S, 415-E/2427/5-2019, 415-E/2427/7-2019 and; dates of approval March 2019; January 2019, amendment June 2019). Informed written consent was obtained from all participants before their inclusion in this study.

### 2.6. Statistics

The statistical analyses were performed using IBM SPSS Statistics version 27 (IBM Corp., Armonk, NY, USA). Quantitative variables, such as age, left ventricular ejection fraction (LVEF), and biomarker levels (e.g., NT-proBNP), were analyzed primarily as continuous variables to preserve information and statistical power. Normal distribution of parameters was assessed by the Shapiro–Wilk test. Baseline parameters were reported as mean and standard error of the mean, provided that the data followed a normal distribution. Means were compared by Student’s *t*-test. Median and inter-quartile range (IQR) was used if the parameters were not normally distributed. These baseline parameters were compared by the Mann–Whitney U-test.

For normal-distributed variables, the dynamics of biomarker concentrations were assessed by a dependent *t*-test and non-normal-distributed variables were compared by Wilcoxon signed rank test. For categorical variables, the chi-squared test was used. To assess correlations, Pearson’s correlation coefficient or Spearman’s rank correlation coefficient was used. All *p*-values were 2 sided and statistical significance was defined as *p* < 0.05. Linear mixed regression models were used to assess the association between ARNI treatment and changes in biomarkers, adjusting for potential confounders identified based on clinical relevance and previous studies, including age and comorbidities. The proportion of missing data was low, and therefore, no significant impact on the results is expected. The authors used ChatGPT-4 (OpenAI, 2025) to support English language revision. The content was controlled and corrected by the authors.

## 3. Results

### 3.1. Demographics and Baseline Characteristics

The baseline characteristics of 70 patients (mean age 62.3 ± 12.4 years, 30.0% female) are shown in Table 1. The mean LVEF was 29.9% (IQR 36.25; 23.0), with approximately half of the cases originating from ischemic genesis and the other half from dilatative genesis (n = 34/36; 48.6%/51.4%). The majority of patients were receiving adequate HF therapy with angiotensin-converting enzyme inhibitors (ACEI) or angiotensin-1 receptor (ARB) inhibitors (85.7%), betablockers (BB) (85.7%) and mineralocorticoid receptor antagonists (68.6%), respectively, before initiation of ARNI therapy. The median follow-up time was 105 days (IQR 95; 164).

### 3.2. Descriptive Statistics

Initially, a substantial proportion of patients suffered from severe symptoms (47.6%, NYHA III/IV) and had a mean NT-proBNP of 1402 pg/mL (IQR 2636; 475). The mean BMI was 26.7 (IQR 30.9; 23.2) and 29.4% were obese (defined as a BMI of 30 or higher). The mean low-density lipoprotein (LDL) level was 86.0 mg/dL (IQR 98.0; 62.8) whereby 44 patients (58.6%) were on statin therapy. Twelve patients had a prior diagnosis of diabetes mellitus (17.9%) with a mean HbA1c of 6.9% ± 1.1% compared to 5.6% ± 0.45% in non-diabetic patients.

### 3.3. Main Findings

**Figure 1:**
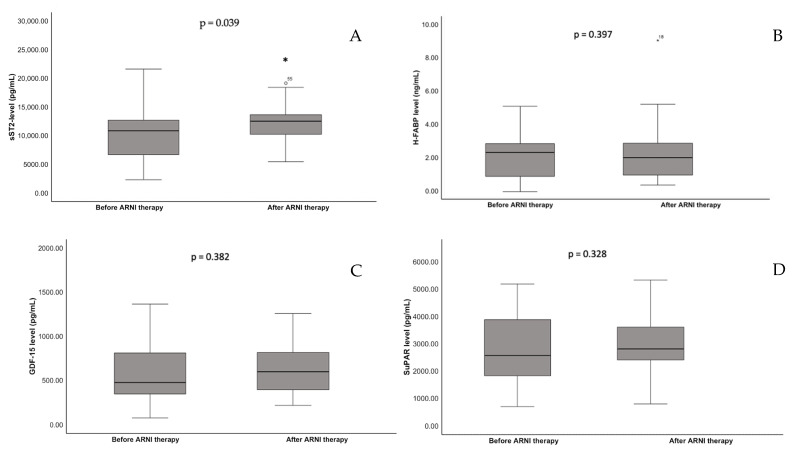
Serum concentrations before and after ARNI therapy of (**A**) sST2, (**B**) H-FABP, (**C**) GDF-15, and (**D**) suPAR. *: *p* < 0.05.

Biomarker dynamics are shown in Figure 1. The mean or median baseline level of sST2 was 9602 pg/mL (SD *±* 4842), that of H-FABP was 2.06 ng/mL (IQR 2.94; 0.90), that of GDF-15 was 956.73 pg/mL (IQR 1000.64; 402.00), and that of suPAR was 3201.30 pg/mL (IQR 4142.82; 2050.36).

Under HF medical therapy, NT-proBNP levels significantly improved to 572.0 pg/mL (IQR 1733.5; 215.3, *p* = 0.003) and the LVEF improved significantly to 38.5% (IQR 50.0; 33.25, *p* < 0.001). Interestingly, along with that, we found a significant increase in sST2 levels to 12,001 pg/mL (SD *±* 5495, *p* = 0.039) but no significant change in H-FABP from 2.06 ng/mL (IQR 2.94; 0.90) to 3.47 ng/mL (IQR 2.94; 0.94, *p* = 0.397), GDF-15 from 956.73 pg/mL (IQR 1000.64; 402.00) to 630.44 pg/mL (IQR 855.91; 362.40, *p* = 0.382) and suPAR from 3201.30 pg/mL (IQR 4142.82; 2050.36) to 3025 pg/mL (IQR 3391; 2334, *p* = 0.328). A linear mixed model was used to adjust the changes in sST2 for CRP as a marker for inflammation and for renal function measured with GFR as confounders. sST2 changes remained significant after correction (*p* = 0.009).

Bivariate analysis showed a significant, strong positive correlation of changes in NT-proBNP with changes in sST2 (*r* = 0.704, *p* < 0.001) and changes in GDF-15 (*r* = 0.743, *p* < 0.001) but no significant correlation with changes in H-FABP (*r* = 0.384, *p* = 0.77) or with changes in suPAR (*r* = 0.108, *p* = 0.632). There was a strong positive correlation of changes in GDF-15 with changes in sST2 (*r* = 0.918, *p* < 0.001) and with changes in H-FABP (*r* = 0.557, *p* = 0.005) but no correlation with changes in suPAR or with other biomarkers. The change in suPAR was the only biomarker significantly correlating with the baseline CRP level (*r* = 0.566, *p* = 0.022). The age did not correlate with changes in sST2 (*r* = 0.070, *p* = 0.713), H-FABP (*r* = −0.052, *p* = 0.806) GDF-15 (*r* = 0.039, *p* = 0.848) or suPAR (*r* = −0.072, *p* = 0.319) in our trial.

**Figure 2:**
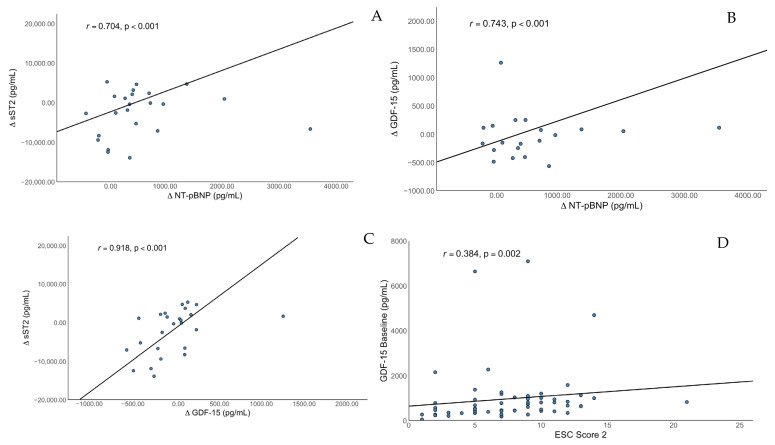
Correlation of change in serum concentration of (**A**) sST2 and NT-proBNP, (**B**) GDF-15 and NT-proBNP, (**C**) sST2 and GDF-15, and (**D**) GDF-15 and ESC Score 2.

In a next step, we calculated the ESC SCORE 2 and found a significant correlation with the baseline sST2 level (*r* = 0.280, *p* = 0.030) and a significant correlation with baseline GDF15 levels (*r* = 0.384, *p* = 0.002). After correction for the confounders age, GFR, underlying cardiomyopathy, atrial fibrillation and diabetes, there was a persistent significant association between baseline sST2 and ESC_SCORE2 (*r* = 0.323, *p* = 0.039). There was also a significant correlation with GDF-15 at follow-up (*r* = 0.504, *p* = 0.011); after correction for confounder (GFR, underlying cardiomyopathy, atrial fibrillation, and diabetes), we found an improvement in the correlation coefficient (*r* = 0.782, *p* = 0.08). No significant correlations with sST2 levels at follow-up (*r* = 0.305, *p* = 0.107), with H-FABP at baseline (*r* = 0.103, *p* = 0.436) and follow-up (*r* = 0.104, *p* = 0.622) or with suPAR at baseline (*r* = 0.034, *p* = 0.794) and follow-up (*r* = −0.053, *p* = 0.801) were found. Correlations are shown in Figure 2.

To evaluate the correlation of the biomarkers with right ventricular function, we calculated the Tei index/MPI, an echocardiographic parameter of right ventricular systolic and diastolic function, at baseline, which correlated with the follow-up levels of sST2 (*r* = −0.762, *p* = 0.028) and GDF15 (*r* = 0.801, *p* = 0.015) but not with the baseline levels (r = −0.154, *p* = 0.585 and *r* = 0.282, *p* = 0.308). The association with GDF-15 was persistent after correction for the confounders age, GFR, atrial fibrillation, and diabetes (*r* = 0.685, *p* = 0.029) and with sST2 (*r* = −0.709, *p* = 0.022).

There was no correlation with H-FABP at baseline (*r* = −0.389, *p* = 0.152) or follow-up (*r* = 0.100, *p* = 0.770) or with suPAR at baseline (*r* = −0.068, *p* = 0.810) or follow-up (*r* = 0.160, *p* = 0.491).

## 4. Discussion

The biomarkers sST2, GDF-15, H-FABP and suPAR showed promising results in multiple cardiovascular diseases. The purpose of this study was to investigate the dynamics of these biomarkers during the novel medical therapy with ARNIs.

sST2 is known to be elevated in chronic HF and is released during inflammatory conditions. Although improvement in HF goes along with a decrease in inflammation, we found an increase in sST2. Considering that ARNIs do not directly intervene in the inflammatory component of HF, the data could reflect processes, which do not immediately respond to ARNI therapy in the early phase.

This might be reflected in previous trials with a much higher percentage of comorbidities that have shown a stronger impact of ARNIs on sST2 levels [50]. A different pathway mechanism might be the effect of increased BNP levels on the inflammatory pathway. Several trials describe the effect of natriuretic peptides as endogenous immunomodulators and their effect on interleukins and tumor necrosis factor alpha (TNF-α). Elevated natriuretic peptide levels have been shown to be protective against inflammation in visceral fat in knockout mice with a deactivated natriuretic peptides clearance receptor. Additionally, administration of natriuretic peptides infusions reduced the severity of acute lung injury induced by endotoxins. Furthermore, protective interleukin pathways were upregulated and a reduction in fibrosis was observed. In clinical settings, infusion of BNP in acute heart failure significantly reduced inflammation represented by decrease in IL-6, TNF-α, and CRP [51]. These effects are partly mediated by suppressing the activation of the pro-inflammatory transcription factor NF-κB, ERK1/2 via the NPR-1/cGMP signaling axis, and components of the NLRP3 inflammation pathway in monocytes and regulating T-cells [52]. The effects of inflammation in heart failure is still a field of research that clearly requires more data [53,54].

Another way of action might reflect an increase in mechanical strain resulting from increased activity due to improved LVEF. During increased physical activity, the biomechanical stress and cardiac stretch are elevated. This finding was derived from patients with stable coronary artery disease who participated in high- and moderate-intensity training. During this trial, an increase in sST2 levels at four months after start of the physical activity was found [55]. Considering the multiple potentially counteracting pathways affecting sST2, the clinical interpretation of sST2 should be approached with caution.

Interestingly, we found a significant correlation of sST2 with GDF-15 levels, supporting the theory that the inflammatory component is not sufficiently affected. However, we could not find a significant effect of ARNI therapy on GDF-15. To date, therapies targeting the inflammatory pathway based on the cytokine hypothesis are rare although beneficial effects of anti-IL-1 β and anti-IL-6 therapy are reported [22]. Considering the complexity of inflammatory processes, it appears more reasonable to target proinflammatory comorbidities with already established therapies. Lichtenauer et al. presented a significant correlation of GDF-15 and sST2 with diabetes [16], which is known to have negative effects on mortality in HF [56]. This might indicate the need of sufficient diabetes therapy in patients with HF.

With respect to the various pathways contributing to heart failure, a multi-biomarker approach to distinguish the etiology of heart failure and assess individual risk represents a potent and promising approach to personalized medicine [16]. The evaluation of dynamics during ARNI therapy in this trial indicates a persistent effect during novel therapeutic agents. In particular, sST2 bears great potential considering its dynamic responsiveness to clinical changes and its resistance to demographic and clinical variables [57]. These results support further evaluation of the differentiation of heart failure entities to gain deeper mechanistic insights and to analyze biomarker-guided therapy in subgroups according to etiology and risk stratification in a larger trial as a next step.

We found a significant correlation of baseline sST2 levels and GDF-15 at follow-up with the ESC Score 2, which is known to be associated with cardiovascular mortality. This outcome aligns with prior studies, confirming the prognostic value of these novel biomarkers [28,29,30]. In the clinical setting, these biomarkers might be a useful tool to determine the cardiovascular risk in patients with HF.

The biomarker sST2 and GDF-15 at follow-up but not H-FABP and suPAR correlated with the Tei index, a marker of right heart function. This further supports the assumption of the interaction of sST2 and GDF-15 in the pathophysiology of HF and their superiority in monitoring HF dynamics over H-FABP and GDF-15.

Taken together, multiple clinical parameters like LVEF, NT-proBNP, ESC Score 2 and MPI/Tei index have been shown to significantly correlate with sST2 and GDF-15, which is a promising result and highlights the potential of these biomarkers in predicting the prognosis in HF patients. Further prospective clinical studies are required to evaluate the potential of sST2 to monitor HFrEF during routine treatment with ARNIs.

## 5. Limitations

To reduce the chance of interactions with our biomarkers, we evaluated for confounders where feasible, but it is important to consider that our results might be affected by other, unknown parameters. During follow-up, no patient was lost, yet two patients had to be excluded due to relevant side effects of ARNIs. It is unlikely that the exclusion had a significant effect on the results but it has to be acknowledged that these data only apply for patients tolerating ARNI therapy. Although a control group was not included due to ethical constraints, we acknowledge that alternative designs—such as pre-post comparisons with multiple time points—could provide additional insights and should be considered in future studies. Considering this was an observational study, some level of selection bias cannot be ruled out. This is a non-randomized trial; therefore, any uncontrolled variables (e.g., lifestyle) bears a residual risk. Regarding the population size, type I and type II errors cannot be ruled out entirely. Although we followed strict inclusion criteria a misclassification bias, e.g., based on interobserver variability, cannot be fully excluded. Due to the limited number of follow-up time points, we could not fully account for time-varying confounding, which may influence biomarker levels and clinical outcomes over the course of treatment.

## 6. Conclusions

This trial was able to highlight the effect of ARNI therapy on novel biomarkers and indicates a superiority of sST2 to act as a biomarker for HF over GDF-15, H-FABP and suPAR with respect to multiple clinical parameters like LVEF, NT-pBNP, ESC Score, and MPI/Tei index. sST2 might be a promising tool in predicting the prognosis in HF patients. Further larger-scale studies are needed to confirm these findings.

## Figures and Tables

**Table 1:** Baseline characteristics; median, lower and upper quartiles or standard deviation (Q1; Q3 or SD) and n (%).

Baseline Characteristics	Specification	Results	Q1; Q3/SD or %
Demographics	Age (years)	62.3	(±12.4)
	Gender (male/female)	49/21	(70.0; 30.0)
Medical history	Ischemic cardiomyopathy	34	(48.6)
	Non-ischemic cardiomyopathy	36	(51.4)
	Atrial fibrillation *	20	(28.6)
	Dyslipidemia	39	(58.2)
	Diabetes mellitus	12	(17.9)
	Hypertension	37	(55.2)
	Chronic kidney disease **	26	(37.0)
	History of smoking	31	(46.2)
Clinical measurement	BMI	26.7	(23.2; 30.9)
	SBP (mmHg)	126	(±18.0)
	Heart rate (bpm)	70	(60; 82)
	LVEF (%)	29.9	(23.0; 36.25)
	LVEDD (mm)	60.2	(±8.6)
Treatment	ACI/ARB (before ARNI)	60	(85.7)
	BB	60	(85.7)
	MRA	48	(68.6)
	Statin	44	(58.6)
	Ezetimib	9	(12.9)
	Loop Diuretics	35	(55.0)
	Thiacids	1	(1.4)
	Metformin	10	(14.3)
	SGLT-2 inhibitors	2	(2.9)
	Insulin	4	(5.7)
Laboratory	NT-proBNP (ng/L)	1402	(475; 2636)
	Hemoglobin g/L	14.0	(±1.9)
	eGFR (mL/min/1.73 m^2^)	64.8	(±16.3)
	LDL (mg/dL)	86.0	(62.8; 98.0)
	HbA1c (%)	5.7	(5.4; 6.1)
	CRP (mg/dL)	0.5	(0.2; 1.4)

* including history of paroxysmal, persistent, or permanent atrial fibrillation; ** eGFR < 60 mL/min/1.73m^2^; BMI: body mass index, SBP: systolic blood pressure, LVEDD: left ventricular end-diastolic ejection fraction, ACI: angiotensin-converting enzyme inhibitor, ARB: angiotensin receptor blocker, BB: betablocker, MRA: mineralocorticoid receptor blocker, SGLT-2: sodium–glucose cotransporter-2 inhibitor, eGFR: estimated glomerular filtration rate, LDL: low-density lipoprotein, CRP: C-reactive protein.

## Data Availability

The data presented in this study are available on request from the corresponding author.

## Supplementary Materials

The following supporting information can be downloaded at: https://www.mdpi.com/article/10.3390/jcm14165668/s1, STROBE Checklist.
