# Supplementary material for: Dynamics of the Novel Cardiac Biomarkers sST2, H-FABP, GDF-15 and suPAR in HFrEF Patients Undergoing Heart Failure Therapy, a Pilot Study
Source: J Clin Med. 2025 Aug 11;14(16):5668. doi: 10.3390/jcm14165668 (PMC12386204; doi:10.3390/jcm14165668)
Supplement: Supplementary file 1 [file jcm-14-05668-s001.zip › jcm-3752780-supplementary.pdf]

STROBE Statement—Checklist of items that should be included in reports of ***cohort studies***

	Item No	Recommendation	Page/Section
**Title and abstract**	1	(*a*) Indicate the study's design with a commonly used term in the title or the abstract	Page 1, Abstract
		(*b*) Provide in the abstract an informative and balanced summary of what was done and what was found	Page 1, Abstract – (2) Methods, (3) Results
**Introduction**			**-**
Background/rationale	2	Explain the scientific background and rationale for the investigation being reported	Page 2 – 4 Introduction
Objectives	3	State specific objectives, including any prespecified hypotheses	Page 4 - Introduction
**Methods**			**-**
Study design	4	Present key elements of study design early in the paper	Page 4 – Methods – 2.1. Study population
Setting	5	Describe the setting, locations, and relevant dates, including periods of recruitment, exposure, follow-up, and data collection	Page 4 – Methods – 2.1. Study population, 2.2. Blood sampling, 2.3. Biomarker analysis
Participants	6	(*a*) Give the eligibility criteria, and the sources and methods of selection of participants. Describe methods of follow-up	Page 4 – Methods - 2.1. Study population
		(*b*) For matched studies, give matching criteria and number of exposed and unexposed	-
Variables	7	Clearly define all outcomes, exposures, predictors, potential confounders, and effect modifiers. Give diagnostic criteria, if applicable	Page 4 – Methods - 2.1. Study population, 2.5. Statistics
Data sources/ measurement	8*	For each variable of interest, give sources of data and details of methods of assessment (measurement). Describe comparability of assessment methods if there is more than one group	Page 4-5 – Methods – 2.1. Study population, 2.2. Blood sampling, 2.3. Biomarker analysis
Bias	9	Describe any efforts to address potential sources of bias	Page 4 – Methods 2.2. Data collection
Study size	10	Explain how the study size was arrived at	Page 4 – Methods 2.2. Data collection
Quantitative variables	11	Explain how quantitative variables were handled in the analyses. If applicable, describe which groupings were chosen and why	Page 6 – Methods 2.6. Statistics
Statistical methods	12	(*a*) Describe all statistical methods, including those used to control for confounding	Page 6 – Methods 2.6. Statistics
		(*b*) Describe any methods used to examine subgroups and interactions	Page 6 – Methods 2.6. Statistics
		(*c*) Explain how missing data were addressed	Page 6 – Methods 2.6. Statistics
		(*d*) If applicable, explain how loss to follow-up was addressed	- (no loss of follow-up)
		(*e*) Describe any sensitivity analyses	Page 6 – Methods 2.6. Statistics
**Results**			**-**

Participants	13*	(a) Report numbers of individuals at each stage of study—eg numbers potentially eligible, examined for eligibility, confirmed eligible, included in the study, completing follow-up, and analysed	Page 4 – Methods – 2.2. Data collection, no loss of follow up
		(b) Give reasons for non-participation at each stage	-
		(c) Consider use of a flow diagram	-
Descriptive data	14*	(a) Give characteristics of study participants (eg demographic, clinical, social) and information on exposures and potential confounders	Page 4 – Methods – 2.1. Study population<br>Page 6 – Results and Table 3.1. Baseline characteristics
		(b) Indicate number of participants with missing data for each variable of interest	Page 6 – Results and Table 3.1. Baseline characteristics
		(c) Summarise follow-up time (eg, average and total amount)	Page 6 – Results – 3.1. Study population
Outcome data	15*	Report numbers of outcome events or summary measures over time	Page 7 - 8 – Results – 3.3. Main findings
Main results	16	(*a*) Give unadjusted estimates and, if applicable, confounder-adjusted estimates and their precision (eg, 95% confidence interval). Make clear which confounders were adjusted for and why they were included	Page 7 - 8 – Results – 3.3. Main findings
		(*b*) Report category boundaries when continuous variables were categorized	-
		(*c*) If relevant, consider translating estimates of relative risk into absolute risk for a meaningful time period	-
Other analyses	17	Report other analyses done—eg analyses of subgroups and interactions, and sensitivity analyses	Page 7 - 8 – results – 3.3. Main findings
**Discussion**			
Key results	18	Summarise key results with reference to study objectives	Page 10-11 – 4. Discussion
Limitations	19	Discuss limitations of the study, taking into account sources of potential bias or imprecision. Discuss both direction and magnitude of any potential bias	Page 12 – 5. Limitations
Interpretation	20	Give a cautious overall interpretation of results considering objectives, limitations, multiplicity of analyses, results from similar studies, and other relevant evidence	Page 11 – 4. Discussion
Generalisability	21	Discuss the generalisability (external validity) of the study results	Page 12 – 6. Conclusion
**Other information**			
Funding	22	Give the source of funding and the role of the funders for the present study and, if applicable, for the original study on which the present article is based	Page 12 Fundings

*Give information separately for exposed and unexposed groups.
